# Bone phenotype in male and female mice after knockdown of transferrin receptor 1 in osterix-expressing cells

**DOI:** 10.1093/jbmrpl/ziaf069

**Published:** 2025-05-23

**Authors:** Vanessa Passin, Maria G Ledesma-Colunga, Ulrike Baschant, Lorenz C Hofbauer, Martina Rauner

**Affiliations:** Department of Medicine III and Center for Healthy Aging, Medical Faculty and University Hospital Carl Gustav Carus, Dresden University of Technology, 01307 Dresden, Germany; Department of Medicine III and Center for Healthy Aging, Medical Faculty and University Hospital Carl Gustav Carus, Dresden University of Technology, 01307 Dresden, Germany; Department of Medicine III and Center for Healthy Aging, Medical Faculty and University Hospital Carl Gustav Carus, Dresden University of Technology, 01307 Dresden, Germany; Department of Medicine III and Center for Healthy Aging, Medical Faculty and University Hospital Carl Gustav Carus, Dresden University of Technology, 01307 Dresden, Germany; Department of Medicine III and Center for Healthy Aging, Medical Faculty and University Hospital Carl Gustav Carus, Dresden University of Technology, 01307 Dresden, Germany

**Keywords:** bone homeostasis, Tfr1, osteoblast, osteoclast, iron

## Abstract

Transferrin receptor 1 (Tfr1) plays a key role in mediating the cellular uptake of transferrin-bound iron. While Tfr1 is essential for iron uptake in erythroid cells and skeletal muscle, it is dispensable for iron acquisition in hepatocytes, intestinal epithelial, or endothelial cells. In this study, we investigated the significance of Tfr1 for iron uptake and cellular function in bone-forming osteoblasts. Therefore, we examined the bone characteristics of male and female Tfr1^fl/fl^;Osx:cre^+/−^ (osteoprogenitors) conditional KO mice at 12 and 24 wk of age. Bone marrow-derived cells from Tfr1^fl/fl^;Osx:cre^+/−^ mice were differentiated into osteoblasts in vitro to assess cellular iron status as well as cellular differentiation and function. Our findings indicate that Tfr1 deficiency in osteoprogenitors in male mice resulted in increased trabecular bone mass in the axial skeleton with decreased bone formation rate as well as decreased levels of serum bone turnover markers. Despite increased bone mass in the femur in females resulting from Tfr1 deficiency in osteoprogenitors, loss of bone mass following ovariectomy was not mitigated. Transferrin receptor 1-deficient osteoblasts showed mild changes in cytosolic iron levels and decreased mineralization. These results suggest a minor role of Tfr1 in osteoblasts differentiation and function but highlight distinct strategies for iron acquisition employed by bone cells to maintain cellular iron homeostasis.

## Graphical Abstract

**Graphical Abstract:**
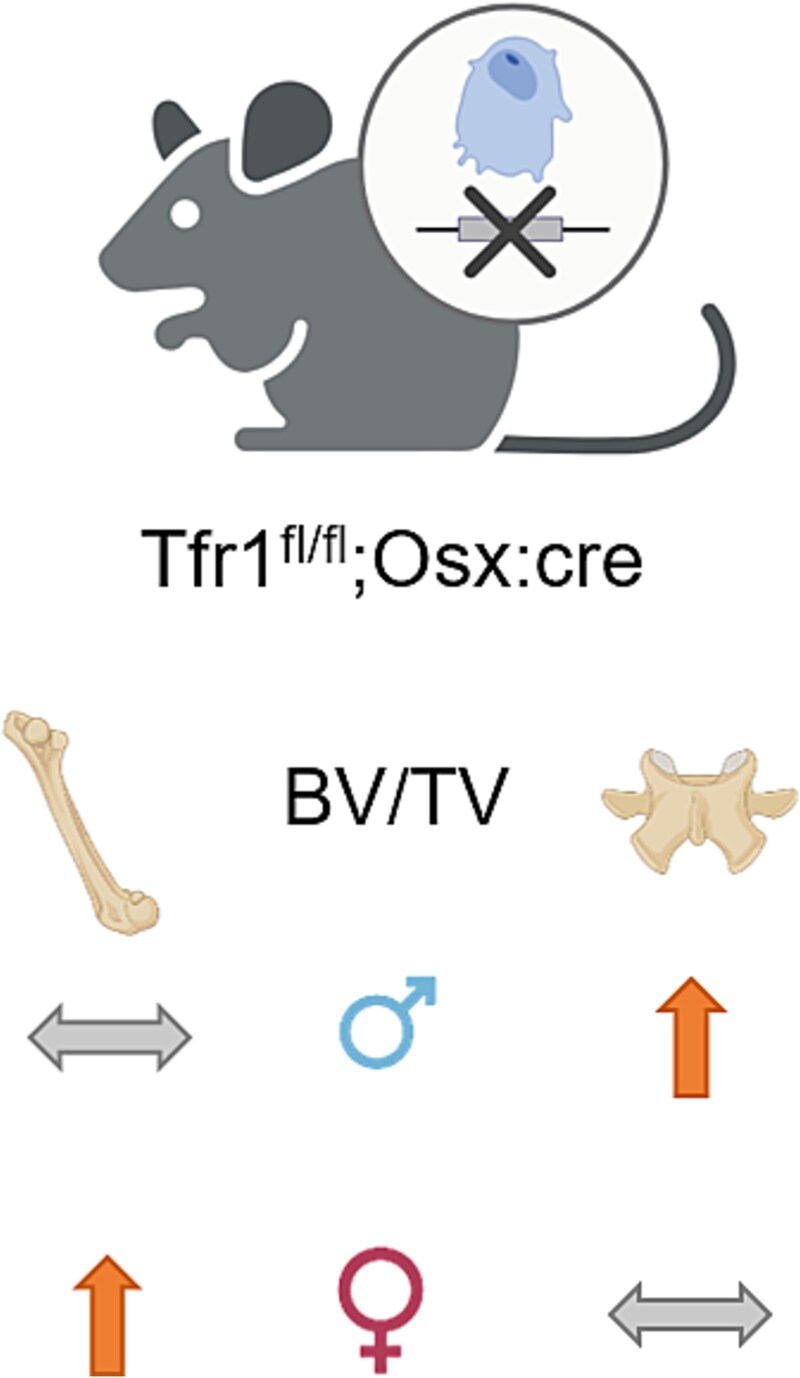

## Introduction

Iron is an essential element for life and is required for various physiological processes. Iron circulates in the body primarily bound to the carrier protein transferrin, ensuring a stable, nonreactive state and facilitating its delivery to tissues. Transferrin mediates high-affinity but pH-reversible binding of ferric iron (Fe^3+^). Transferrin receptor 1 (Tfr1, encoded by *Tfrc* gene) plays a key role in mediating the cellular uptake of transferrin-bound iron. Transferrin receptor 1 internalizes transferrin-bound iron by receptor-mediated endocytosis, followed by the release of iron in the endosome and subsequent transport into the cytoplasm, where it can be further used for cellular processes, stored, or exported. Transferrin receptor 1 and apo-transferrin are recycled back to the cell surface to mediate further rounds of iron uptake.^1^ Cellular iron acquisition apart from Tfr1 has been described in the form of nontransferrin bound iron (NTBI) via noniron-specific metal transporters (eg, Zip8/14, Dmt1)^2^ or in some cell types in the form of heme-iron or ferritin through specified transporters (eg, Flvcr2, Hcp1, Scara5).^3–6^

Global or tissue-specific disruption of Tfr1 has shown that Tfr1-mediated iron uptake is a major iron acquisition pathway for some, but not all cell types. Transferrin receptor 1 is crucial for hematopoiesis as observed in heterozygous Tfr1 KO mice, which show microcytic hypochromic erythrocytes,^7^ and in mice lacking Tfr1 in the hematopoietic compartment, which die within 1 wk after birth due to insufficient erythropoiesis.^8^ In addition, nonhematopoietic cells such as cardiomyocytes,^9^ dopaminergic neurons,^10^ and skeletal muscle cells^11^ have been shown to critically depend on Tfr1 for iron acquisition. The role of Tfr1 in T-cells during adaptive immunity^12^ and for thermogenic regulation in adipocytes^13^^,^^14^ has also been described. Although Tfr1 is ubiquitously expressed, some tissues do not rely exclusively on Tfr1-mediated iron uptake. Knockout of Tfr1 in hepatocytes^15^ and liver endothelial cells^16^ did not impact cellular iron supply but highlighted alternate iron acquisition pathways employed by these cell types. Apart from iron uptake, Tfr1 has been described to be involved in signaling activities,^17^^,^^18^ in fine-tuning hepcidin expression,^15^^,^^19^ and maintaining intestinal tissue homeostasis.^20^

Bone homeostasis and proper bone cell function depend on balanced systemic and cellular iron concentrations.^21^ The mechanisms of how iron is taken up by bone cells remain incompletely understood. Studies have shown that Tfr1 expression is upregulated during osteoclast differentiation and is required for mitochondrial biogenesis.^22^^,^^23^ Treatment of osteoclasts with holo-transferrin stimulated their formation in a dose-dependent manner and was prevented by knockdown of Tfr1.^22^ Recently, the role of Tfr1-mediated iron uptake in osteoclasts has been investigated.^24^ The study also showed that Tfr1 expression is upregulated during osteoclast differentiation and that the loss of Tfr1 in osteoclasts markedly reduced intracellular iron content. Knockdown of Tfr1 in myeloid osteoclast precursors using a double-allele LysM-Cre strategy resulted in a significant increase in trabecular bone mass in long bones of female but not male mice. Mechanistically, Das and colleagues^24^ proposed that loss of Tfr1 led to impaired mitochondrial function and actin organization resulting in decreased bone resorption with no impact on osteoclast differentiation. These studies highlight the potential importance of Tfr1 for bone homeostasis. However, the relevance of Tfr1 for iron uptake in osteogenic cells as well as its relevance for bone cell function and homeostasis has not been conclusively addressed.

In this study, we validated the bone phenotype of the Tfr1^fl/fl^;LysM:cre^+/−^ and explored the significance of Tfr1 for cellular function of osteoblasts generating cell-specific Tfr1^fl/fl^;Osx:cre^+/−^ conditional KO mice and assessed bone microarchitecture as well as bone cell function and cellular iron status in vitro. Our findings revealed that Tfr1 deficiency in osteoprogenitors led to a site- and sex-specific increase in trabecular bone parameters in vivo and only minor changes in osteoblast function in vitro. Our study suggests that, like other cell types, osteoblasts may also employ additional strategies for iron uptake. Further analysis of iron acquisition in bone cells is necessary to enhance our understanding of mechanisms involved in bone loss under iron imbalance conditions.

## Materials and methods

### Animal models

*Tfrc* ^fl/fl^ mice were originally produced and kindly provided by Dr Kostas Pantopoulos (McGill University).^15^ The Osx:cre and LysM:cre transgenic mouse lines as well as the C57BL/6J mice were obtained from The Jackson Laboratory (Bar Harbor). For a cell-specific deletion of Tfr1, Tfr1^fl/fl^;LysM:cre (osteoclast precursors, Tfr1^fl/fl^;LysM:cre^+/−^) and doxycycline-repressible Tfr1^fl/fl^;Osx:cre (osteoprogenitors, Tfr1^fl/fl^;Osx:cre^+/−^) were generated. Tfr1^fl/fl^;Osx:cre breeding pairs continuously received doxycycline in their drinking water (10 mg/mL in 30% sucrose solution) to repress cre-recombinase activity during embryogenesis. Offspring was maintained on doxycycline-containing water until 6 wk of age.^25^ Respective cre-negative littermates (Tfr1^fl/fl^;LysM:cre^−/−^, Tfr1^fl/fl^;Osx:cre^−/−^) were used as controls.

Genomic DNA from ear biopsies was isolated and further used for genotyping by PCR. The following primers were used: *Cre s*: CGGTCGATGCAACGAGTGATGAGG; *Cre as*: CCAGAGACGGAAATCCATCGCTCG; *Tfr1-flox s*: CCCAAGTGCTGGAGATTGTAG; *Tfr1-flox as*: AGCTGTCTTCAAACACACCAGA.

All animal procedures were conducted in compliance with the faculty guidelines on Animal Welfare and were approved by Landesdirektion Sachsen, Germany (TVV 7/2022, TVV 20/2020, TVT 3/2023). Mice were maintained in groups of up to 5 animals per cage under a 12 hr dark/light cycle at a controlled temperature (23 °C) with food (standard chow containing 176 mg iron/kg, Ssniff V1534-3) and water ad libitum until sacrifice at 12 or 24 wk of age.

### Ovariectomy

Ten-week-old female mice (12 mice per genotype) were randomly assigned to 2 groups: control (Sham) and ovariectomy (Ovx). Mice were anesthetized with an intraperitoneal injection of a combination of ketamine (0.1 mg/g of body weight) and xylazine (0.01 mg/g of body weight). For Ovx, bilateral dorsal incisions were made to remove both ovaries after ligating blood vessels. Sham animals received the same procedure but without ligation and removal of the tissue. Mice received metamizole sodium monohydrate (Lichtenstein, Zentiva) in their drinking water for 7 d and were sacrificed for further analysis 4 wk postsurgery.^26^

### Analysis of bone structure and histomorphometry

Bone microarchitecture from excised femora and the fourth lumbar vertebrae (L4) was analyzed using the vivaCT 40 (Scanco Medical) at an isotropic voxel size of 10.5 μm (70 kVp, 114 μA, and 200 ms integration time). The trabecular bone compartment at the distal femur and mid-vertebrae was isolated by manual contouring, and each compartment included regions spanning 100 slices. For analysis, established protocols from Scanco Medical were applied to assess trabecular parameters such as bone volume per total volume (BV/TV), trabecular number (Tb.N), thickness (Tb.Th), and separation (Tb.Sp). Analysis of cortical bone comprised analysis of the cortical thickness (Ct.Th) at the femoral midshaft (using 150 slices). The results are reported following international guidelines.^27^

For dynamic bone histomorphometry, mice received intraperitoneal injections of calcein (20 mg/kg, Sigma) on days 5 and 2 before sacrifice. Dissected vertebrae were fixated in 4% paraformaldehyde for 48 hr and dehydrated in ascending ethanol series. The third to fourth lumbar vertebral body was embedded in methyl methacrylate (Technovit). Fluorescence double labeling was assessed at 7 μm sections to determine the bone formation rate per bone surface (BFR/BS). To assess the number of osteoclasts per bone perimeter (N.Oc/B.Pm), tartrate-resistant acid phosphatase (TRAP) staining was performed on 2 μm paraffin sections of decalcified third vertebral bodies. All measurements were performed using the Osteomeasure software (OsteoMetrics).

### Blood and serum measurements

Blood was obtained from heart puncture of anesthetized mice. Blood parameters were measured using an automated cell counter (Sysmex). Blood was further processed by centrifugation for 20 min at 1000 g to collect serum for further analysis. N-terminal propeptide of type I procollagen (PINP) and TRAP form 5b (TRAcP5b) as markers of bone formation and bone resorption were measured with ELISA (Immunodiagnostic Systems) according to the manufacturer’s protocol. Serum iron concentration was determined using the IRON (SFBC) Bathophenanthrolin kit (Biolabo), and unsaturated iron-binding capacity (UIBC) was measured with the UIBC kit (Biolabo). Transferrin saturation was calculated with the formula: (SFBC/(SFBC + UIBC)) × 100.

### Determination of tissue iron content

Small pieces of liver were collected, dried at 37 °C for 72 hr, and incubated by shaking (300 rpm) in 10% trichloroacetic acid/10% hydrochloric acid in distilled water for 48 hr at 65 °C. Nonheme iron content was determined using the bathophenanthroline colorimetric method.^28^ Iron content is reported as mg iron/g of dry tissue weight calculated based on serial dilution of ferric iron standard (Sigma-Aldrich).

### Osteoclast and osteoblast cell culture

For Tfr1^fl/fl^;LysM:cre osteoclast cell culture, bone marrow cells were flushed from femora and tibiae and seeded in α-MEM (Sigma-Aldrich) supplemented with 10% FCS (Bio&Sell), 1% penicillin/streptomycin (Gibco), 1% L-alanyl-L-glutamine (Sigma-Aldrich) and 10 ng/mL recombinant macrophage colony-stimulating factor (rM-CSF, R&D Systems) in a 75 cm^2^ cell culture flask for 24 hr. Nonadherent cells were collected and seeded in α-MEM containing 10% FCS, 1% penicillin/streptomycin, 1% L-alanyl-L-glutamine, and 25 ng/mL rM-CSF. After 3 d, the medium was further supplemented with 50 ng/mL of recombinant murine soluble RANKL (R&D Systems).

For osteoblast cell culture, bone marrow cells from Tfr1^fl/fl^;Osx:cre or C57BL/6J mice were seeded in DMEM (Gibco) containing 20% FCS and 1% penicillin/streptomycin. At 80% of confluence, cells were switched into osteogenic medium containing DMEM supplemented with 10% FCS, 1% penicillin/streptomycin, 10 mm β-glycerol phosphate, and 100 μm ascorbic acid (both from Sigma-Aldrich) and maintained for 10 or 21 d. Mineralization was assessed by Alizarin Red S staining. Cells were washed with PBS, fixed in 10% paraformaldehyde, and stained with Alizarin Red S solution (Sigma-Aldrich). Excess dye was removed by repeatedly washing the plate with distilled water. The amount of incorporated calcium was eluted with 100 mm cetylpyridinium chloride (Sigma-Aldrich). Eluted dye was transferred to a 96-well plate in duplicates, and absorbance was measured at 540 nm using the FLUOstar Omega microplate reader (BMG Labtech). Values were normalized to cre-negative control cells. Iron treatments of C57BL/6J-derived osteoblasts were performed using 50 μm holo-Transferrin (holo-Tf), ferric ammonium citrate (FAC), or hemin (all from Sigma) in osteogenic media during the differentiation for 21 d.

### RNA isolation, reverse transcription, and quantitative real-time PCR

RNA extraction from cultured osteoblasts or osteoclasts was performed using the RELIA Prep Kit (Promega) according to the manufacturer’s protocol. RNA from flushed bones was isolated using Trizol (Invitrogen), which was crushed in liquid nitrogen using a mortar and pestle. Complementary DNA was synthesized from 1 μg of RNA by reverse transcription using random primers (Thermo Fisher Scientific), dNTPs (Roth), M-MLV Reverse Transcriptase, and RNasin (both from Promega). mRNA expression was assessed by quantitative real-time PCR (ABI 7500Fast, Applied Biosystems) using 20 ng of cDNA, GoTaq Mastermix (Promega), and 10 μm of respective primers (Table S1). Results were calculated using the 2^−∆∆Ct^ method relative to the housekeeping gene *Actb*.

### Calcein-AM measurement

The Calcein-AM method was used to measure cytosolic iron levels. Calcein fluorescence is quenched by chelation of labile iron so that the degree of quenching provides an estimation of the amount of chelatable cytosolic iron.^29^ Osteoblasts were seeded in black-walled 96-well plates (Greiner). On day 10 of differentiation, cells were washed with HBSS, and 5 μm Calcein-AM (BD Biosciences) in PBS supplemented with 20 mm HEPES and 1 mg/mL BSA was added. After 30 min of incubation at 37 °C, fluorescence was measured with the FLUOstar Omega microplate reader (BMG Labtech).

### Protein isolation and western blotting

Protein lysates from osteoblasts and osteoclasts were prepared using RIPA buffer (Sigma-Aldrich) supplemented with protease inhibitor and phosphatase inhibitor (Roche). Cell membranes were disrupted by sonication, and supernatant was collected after centrifugation. Quantification of protein content was performed using the Pierce BCA protein assay kit (Thermo Scientific). Twenty-five microgram of protein was denatured at 95 °C, separated by SDS-PAGE, and transferred onto a nitrocellulose membrane using a semidry blotter (Biorad). After blocking the membrane in 5% BSA, the primary antibody was applied and incubated overnight at 4 °C followed by incubation with HRP-linked secondary antibody (Cell Signaling) for 1 hr. Chemiluminescent detection was performed using Pierce ECL western blotting substrate (Thermo Scientific). The following antibodies were used: anti-GAPDH (1:1000, HyTech, 5G4), Anti-Ferritin Heavy Chain (1:1000, Abcam, ab183781), Anti-Ferroportin 1 (1:1000, Alpha Diagnostic, MTP11-A). Band intensities were quantified by ImageJ (National Institutes of Health). Results were calculated relative to GAPDH.

### Statistical analysis

Results are presented as mean ± SD. The ROUT test (*Q* = 1) was employed to detect and remove outliers (max. 1). For the comparison of 2 groups, an unpaired Student’s *t*-test was performed. For experiments with 2 factors, 2-way ANOVA followed by Bonferroni post hoc test was performed using GraphPad Prism version 10.0.0 for Mac, GraphPad Software, www.graphpad.com. *p*-values <.05 were considered statistically significant. Each dot represents individual mouse data.

## Results

### Partial deletion of Tfr1 in osteoclast precursor cells has no major impact on bone microstructure

Transferrin receptor 1-mediated iron uptake is a main iron acquisition pathway for osteoclasts and is important for their function and bone remodeling.^22^^,^^24^ We have generated macrophage-specific Tfr1 KO mice (Tfr1^fl/fl^;LysM:cre^+/−^) and assessed their bone microarchitecture at 12 and 24 wk of age and compared it to cre-negative littermate controls (Tfr1^fl/fl^;LysM:cre^−/−^). As previously reported,^24^ macrophage-specific Tfr1 KO mice with a single Cre-allele (Tfr1^fl/fl^;LysM:cre^+/−^) only display a partial deletion of Tfr1 in macrophages (−80%, *p* < .001 vs Tfr1^fl/fl^;LysM:cre^−/−^) and osteoclasts (−50%, *p* < .001 vs Tfr1^fl/fl^;LysM:cre^−/−^). Neither young nor adult male or female myeloid cell-specific Tfr1-deficient mice exhibited changes in body weight and blood parameters. Liver iron content as well as transferrin saturation of 12-wk-old Tfr1^fl/fl^;LysM:cre^+/−^ females was significantly increased but normalized at 24 wk of age (Table S1).

According to previously published results,^24^ male Tfr1^fl/fl^;LysM:cre^+/−^ mice displayed no significant alterations in bone microarchitecture at 12 and 24 wk of age, except a slight reduction in bone volume at 12 wk of age (Figure S1A and B). No changes in the levels of the bone formation marker PINP were detected in the cre-positive animals. Bone resorption marker TRAcP5b showed a significant decrease at 12 wk of age, which was not apparent at 24 wk (Figure S1C and D). Furthermore, Tfr1 deficiency did not alter the bone formation rate as well as the number of osteoclasts in the fourth vertebral body (Figure S1E and F). Female Tfr1^fl/fl^;LysM:cre^+/−^ mice displayed no improvement in bone microarchitecture after Ovx (Figure S1G-J). Thus, a partial deletion of Tfr1 in osteoclast precursors does not result in altered bone homeostasis.

### Deficiency of Tfr1 in osteoprogenitors increases axial bone density in male mice

In contrast to the role of Tfr1 in osteoclasts, its role in osteogenic cells is still unknown. Therefore, we depleted Tfr1 from osteoprogenitor cells using Tfr1^fl/fl^;Osx:cre^+/−^ conditional KO mice and assessed the bone microarchitecture of male and female mice at 12 and 24 wk of age and compared it to cre-negative littermate controls (Tfr1^fl/fl^;Osx:cre^−/−^). As in macrophage-specific Tfr1 KO mice, a single Cre-allele (Tfr1^fl/fl^;Osx:cre^+/−^) only results in a partial deletion of Tfr1 in osteoblasts (−50%, *p* < .01 vs Tfr1^fl/fl^;Osx:cre^−/−^, Figure 3A). Female Tfr1^fl/fl^;Osx:cre^+/−^ conditional KO mice did not show any differences in weight, blood cell parameters, or systemic iron parameters compared to cre-negative controls (Table 1). At 12 wk of age, male mice exhibited no changes in serum and liver iron content but higher red blood cell counts, hematocrit, and hemoglobin, which resolved again at 24 wk of age (Table 1). Cre-positive mice developed similarly to littermate controls, but only male cre-positive mice at 24 wk of age showed a significantly lower body weight compared to littermate controls (Table 1).

**Table 1:** Blood and iron parameters of male and female 12- and 24-wk-old Tfr1^fl/fl^;Osx:cre mice.

**Parameter**	**Male**			**Female**		
	**Cre−**	**Cre+**	***p*-value**	**Cre−**	**Cre+**	***p*-value**
**Tfr1** ^**fl/fl**^**;Osx:cre**						
**12 wk**	*n =* 11	*n* = 12		*n* = 11	*n* = 12	
**Body weight (g)**	26.8 ± 2.83	25.8 ± 1.88	.487	21.6 ± 1.37	20.8 ± 1.14	.203
**RBC (×10**^**6**^**/μL)**	9.4 ± 1.15	10.4 ± 0.96	**.032**	9.6 ± 0.74	10.0 ± 0.58	.149
**HGB (mmol/L)**	7.5 ± 0.91	8.5 ± 0.77	**.013**	8.2 ± 0.54	8.4 ± 0.48	.398
**HCT (L/L)**	0.40 ± 0.05	0.47 ± 0.05	**.005**	0.43 ± 0.03	0.45 ± 0.03	.279
**MCV (fL)**	42.8 ± 0.91	44.8 ± 3.09	.067	45.2 ± 3. 1	44.6 ± 2.69	.649
**MCH (fmol)**	0.80 ± 0.03	0.82 ± 0.06	.364	0.85 ± 0.06	0.84 ± 0.05	.473
**Plasma Fe (μg/dL)**	334.2 ± 162.2	306.6 ± 100.7	.715	284.4 ± 69.9	276.6 ± 47.7	.776
**Tf-saturation (%)**	53.6 ± 9.0	51.1 ± 12.9	.689	52.5 ± 14.1	58.7 ± 13.8	.334
**Liver iron (μg/g of dry tissue)**	216.2 ± 233.3	168.4 ± 57.3	.518	354.4 ± 94.0	357.4 ± 148.4	.957
**24 wk**	*n* = 13	*n* = 10		*n* = 10	*n* = 9	
**Body weight (g)**	30.0 ± 3.2	26.7 ± 3.7	**.029**	21.9 ± 1.4	22.6 ± 3.4	.583
**RBC (×10**^**6**^**/μL)**	9.7 ± 0.79	10.0 ± 1.04	.437	9.8 ± 1.29	9.6 ± 1.44	.767
**HGB (mmol/L)**	8.3 ± 0.76	8.2 ± 0.63	.746	8.5 ± 0.84	8.6 ± 1.13	.846
**HCT (L/L)**	0.45 ± 0.04	0.44 ± 0.04	.697	0.46 ± 0.05	0.46 ± 0.07	.954
**MCV (fL)**	46.9 ± 4.52	44.7 ± 4.03	.266	47.1 ± 4.37	48.2 ± 4.14	.629
**MCH (fmol)**	0.86 ± 0.08	0.83 ± 0.07	.284	0.87 ± 0.08	0.90 ± 0.09	.500
**Plasma Fe (μg/dL)**	230.5 ± 49.3	210.6 ± 56.9	.387	265.1 ± 68.0	217.2 ± 73.8	.196
**Tf-saturation (%)**	45.9 ± 7.7	40.1 ± 7.1	.080	46.8 ± 9.6	39.7 ± 8.5	.164
**Liver iron (μg/g of dry tissue)**	447.1 ± 185.3	373.0 ± 181.8	.332	751.9 ± 200.3	960.7 ± 438.6	.224

Data represent the mean ± SD. Statistical analysis was performed using the Student’s *t*-test. Significant values (*p* < .05) are highlighted in bold text. Abbreviations: HCT, hematocrit; HGB, hemoglobin; MCH, mean corpuscular hemoglobin; MCV, mean corpuscular volume; RBC, red blood cells; Tf-saturation, transferrin saturation.

**Figure 1:**
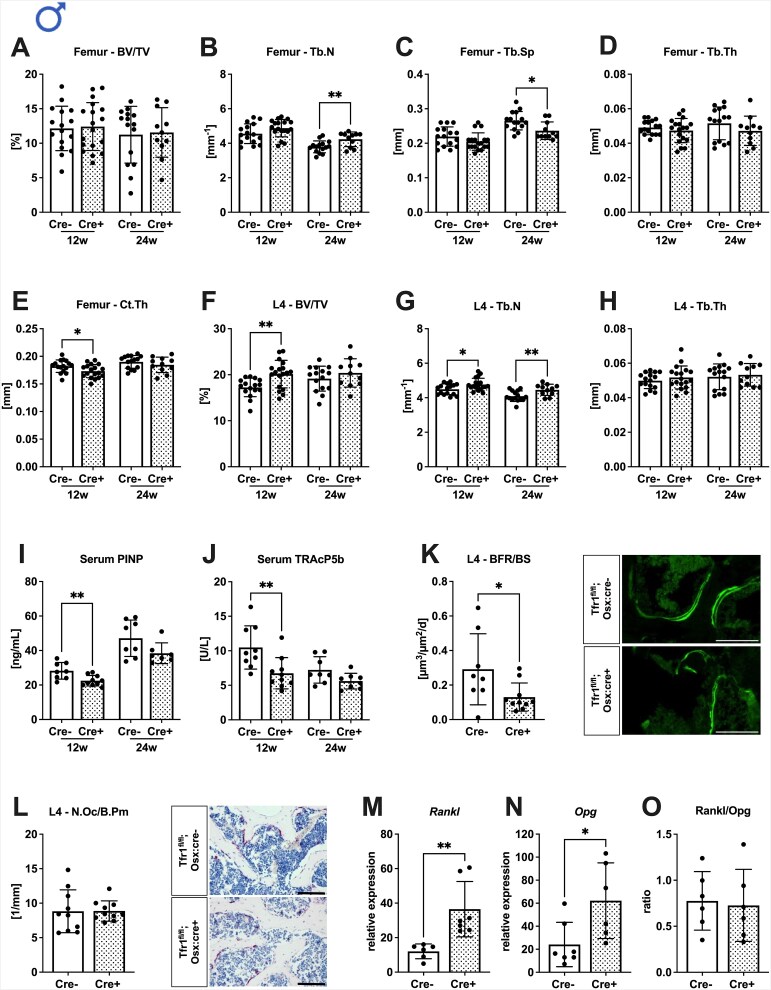
Transferrin receptor 1 deficiency in osteoprogenitors results in increased bone mass and decreased bone turnover in males. Distal femora (A-E) and fourth lumbar vertebrae (F-H) of 12- and 24-wk-old male Tfr1^fl/fl^;Osx:cre mice were analyzed by μCT. Trabecular parameters (A, F) BV/TV, (B, G) trabecular number (Tb.N), (C) trabecular separation (Tb.Sp), (D, H) trabecular thickness (Tb.Th), as well as the cortical parameter (E) cortical thickness (Ct.Th), were assessed. (I, J) Serum levels of PINP and TRAP form 5b (TRAcP 5b) were measured. Histological parameters of the fourth lumbar vertebrae (K, L) were assessed, including (K) BFR/BS and (L) N.Oc/B.Pm. Gene expression levels of (M) Rankl and (N) Opg in bone were analyzed by RT-qPCR, and (O) the Rankl/Opg ratio was formed. Scale bar, 100 μm. Data represents the mean ± SD (*n* = 10-13). Each dot represents an individual mouse. Statistical analysis was performed using the Student’s *t*-test. ^*^p < .05, ^**^p < .01.

Male Tfr1^fl/fl^;Osx:cre^+/−^ mice displayed no significant changes in the trabecular bone microarchitecture in the femur at 12 wk of age, whereas at 24 wk of age, trabecular number was increased, and correspondingly trabecular separation was decreased (Figure 1A-C). Femoral trabecular thickness was unaltered at both ages (Figure 1D). The cortical thickness of 12-wk-old Tfr1^fl/fl^;Osx:cre^+/−^ mice was significantly reduced (Figure 1E). At the axial skeleton, bone volume was increased in younger mice as well as trabecular number at both ages (Figure 1F-G). Vertebral trabecular thickness was unaltered at both ages (Figure 1H). To determine whether changes in bone mass were a result of changed bone turnover, we assessed bone formation by measurement of PINP levels and bone resorption by TRAcP5b levels in the serum. Transferrin receptor 1 deficiency resulted in decreased PINP and TRAcP5b levels in 12-wk-old males, while only tendencies were detected in 24-wk-old mice (Figure 1I-J). Dynamic histomorphometry using calcein labeling revealed a significantly reduced bone formation rate in the vertebrae (Figure 1K); however, the numbers of osteoclasts were unaltered (Figure 1L). Since the bone formation rate and levels of bone turnover markers were lower in cre-positive mice, we measured the expression levels of regulators of bone mass in the bone tissue. We found increased mRNA levels of *Rankl* and *Opg* in the bones of Tfr1-deficient mice, while the Rankl/Opg-Ratio was unaltered (Figure 1M-O).

Of note, also Tfr1^fl/fl^;Bglap:cre^+/−^ (mature osteoblasts) were analyzed for their bone microarchitecture but did not display a significant bone phenotype compared to their cre-negative littermate controls (Table S3).

### Deficiency of Tfr1 in osteoprogenitors does not mitigate Ovx-induced bone loss

**Figure 2:**
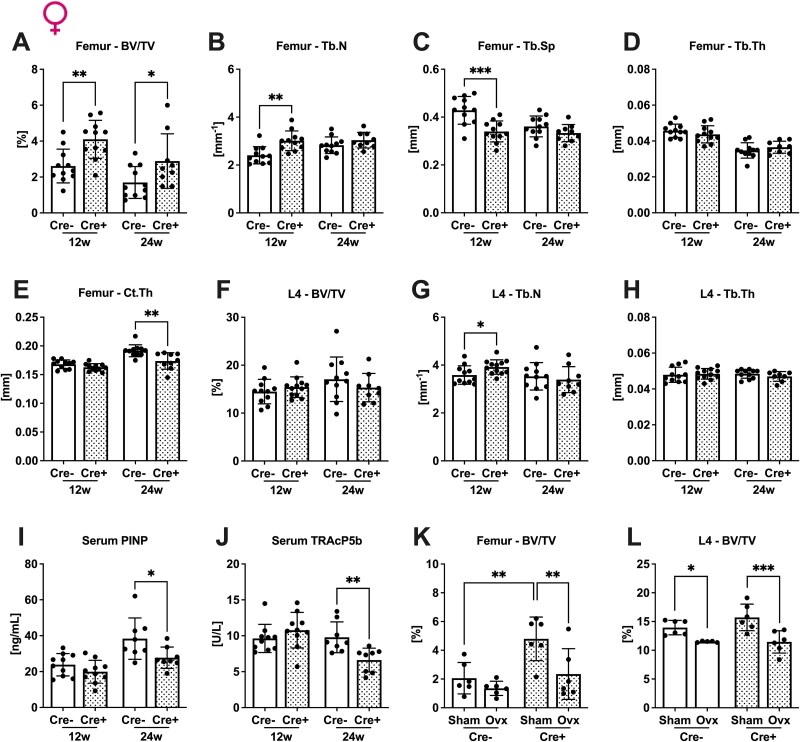
Transferrin receptor 1 deficiency in osteoblasts results in increased bone mass in females and does not rescue OVX-induced bone loss. Distal femora (A-E) and fourth lumbar vertebrae (F-H) of 12- and 24-wk-old female Tfr1^fl/fl^;Osx:cre mice were analyzed by μCT. Trabecular parameters (A, F) BV/TV, (B, G) trabecular number (Tb.N), (C) trabecular separation (Tb.Sp), (D, H) trabecular thickness (Tb.Th), as well as the cortical parameter (E) cortical thickness (Ct.Th), were assessed. (I, J) Serum levels of PINP and TRAP form 5b (TRAcP 5b) were measured. (K, L) Bone volume per total volume of (K) femur and (L) lumbar vertebrae of 14-wk-old mice that underwent Ovx or Sham operation. Data represents the mean ± SD (*n* = 6-12). Each dot represents an individual mouse. For the comparison of 2 groups, a Student’s *t*-test was performed. For experiments with 2 factors, 2-way ANOVA followed (post hoc Bonferroni) was performed. ^*^p < .05, ^**^p < .01, ^***^p < .001.

Female Tfr1^fl/fl^;Osx:cre^+/−^ mice showed a similar phenotype as the male mice, exhibiting increased bone volume, trabecular number, and decreased trabecular separation at the distal femur at 12 wk of age but no significant changes at 24 wk (Figure 2A-C). Femoral trabecular thickness was unaltered at both ages (Figure 2D). Only cortical thickness was reduced in cre-positive females at 24 wk of age (Figure 2E). At the fourth lumbar vertebrae, bone volume remained unchanged, but trabecular number was increased in 12-wk-old cre-positive individuals (Figure 2F and G). Vertebral trabecular thickness was unaltered at both ages (Figure 2H). Transferrin receptor 1 deletion led to a decrease in serum levels of both bone turnover markers (PINP and TRAcP5b) in older females (Figure 2I and J). Finally, we investigated whether the observed increase in femoral bone mass of Tfr1-deficient females could protect from estrogen deficiency-induced bone loss. To address this, we performed bilateral Ovx to mimic postmenopausal bone loss. Transferrin receptor 1 deficiency in osteoprogenitors could not mitigate Ovx-induced bone loss (Figure 2K and L).

Taken together, Tfr1 deficiency in osteoprogenitors resulted in a mild increase in bone mass and decreased bone turnover. While male mice show a stronger bone phenotype in the axial skeleton, female mice exhibit a stronger bone phenotype in the appendicular skeleton.

### Tfr1 deficiency does not majorly alter osteoblast differentiation and function in vitro

**Figure 3:**
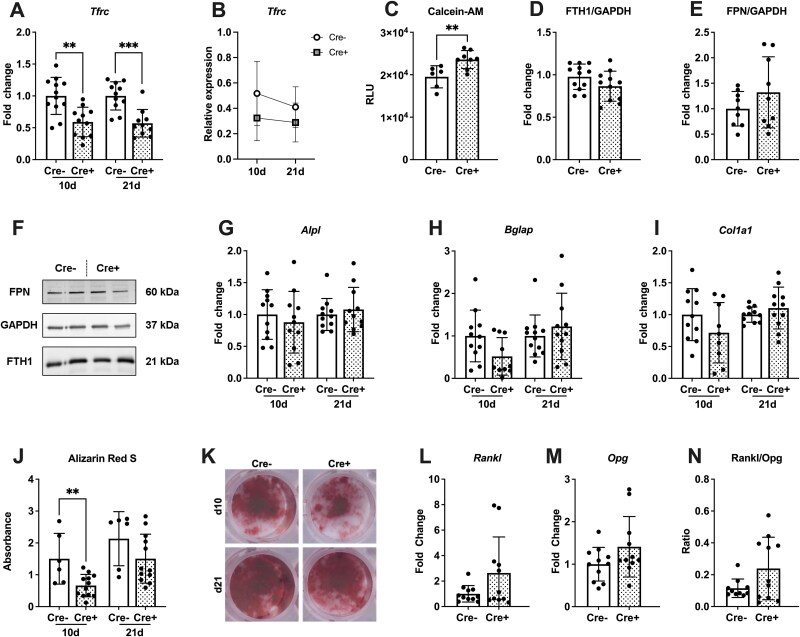
Transferrin receptor 1 deficiency does not majorly alter osteoblast differentiation and function in vitro. Bone marrow-derived cells from Tfr1^fl/fl^;Osx:cre mice were differentiated into osteoblasts for 10 or 21 d. (A) Knockdown of Tfr1 was confirmed by RT-qPCR. (B) Expression levels of *Tfrc* throughout differentiation were determined. (C) Calcein-AM staining was performed to measure cytosolic iron content. (D-F) Protein levels of ferritin heavy-chain and ferroportin were assessed by western blot. (G-I) Gene expression of osteoblast-specific genes *Alpl*, *Bglap*, and *Col1a1* were assessed using RT-qPCR. (J, K) Mineralization of cells was assessed by Alizarin Red S staining. mRNA levels of (L) Rankl and (M) Opg were measured in cells. (N) The Rankl/Opg ratio was formed. Data represent mean ± SD. Statistical analysis was performed using the Student’s *t*-test. Each dot represents an individual mouse. ^**^p < .01, ^***^p < .001.

To further investigate changes in cellular iron status and osteoblast function of Tfr1-deficient osteoblasts, we differentiated primary bone marrow-derived mesenchymal stromal cells into osteoblasts. Knockdown of Tfr1 could be confirmed by gene expression analysis indicating a roughly 50% reduction in Tfr1 levels (Figure 3A). Overall, the expression of Tfr1 decreased over the course of osteoblast differentiation (Figure 3B). We assessed cytosolic iron by measurement of Calcein-AM. Transferrin receptor 1-deficient cells exhibited an increase in the fluorescent signal corresponding to a significantly decreased amount of cytosolic iron (Figure 3C). We performed a Western blot to assess how iron storage protein ferritin and iron exporter ferroportin 1 were altered under Tfr1-deficient conditions.^30^^,^^31^ Protein levels of ferritin as well as ferroportin were not altered by deficiency of Tfr1 in osteoblasts (Figure 3D-F). The mRNA levels of osteoblast-specific genes such as *Alpl*, *Bglap*, or *Col1a1* were unchanged in Tfr1-deficient cells at days 10 and 21 of osteogenic differentiation (Figure 3G-I). However, Tfr1 deficiency in osteoblasts led to a significant decrease in mineralization on day 10 of differentiation, whereas mature osteoblasts on day 21 showed no change compared to control cells (Figure 3J and K). Expression levels of *Rankl* and *Opg* were not significantly increased but showed tendencies (Figure 3L and M). Also, the Rankl/Opg ratio tended to be increased in Tfr1-deficient osteoblasts (Figure 3N).

In summary, Tfr1-deficient osteoblasts showed signs of a mild decrease in cytosolic iron, while their differentiation and mineralization remained largely unaltered.

### Osteoblasts express various iron transport molecules apart from Tfr1

**Figure 4:**
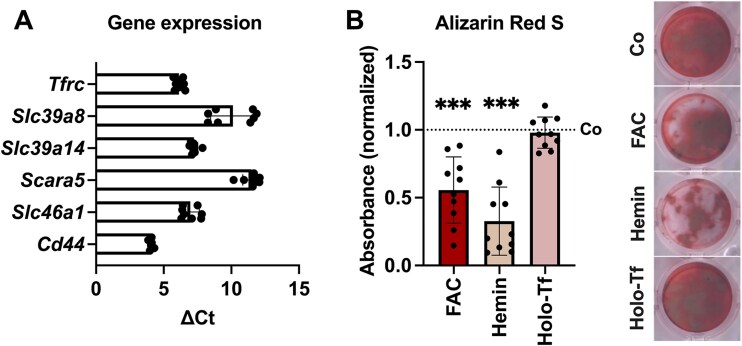
Osteoblasts express various iron transport molecules and show decreased mineralization after NTBI treatment. Bone marrow-derived cells from C57BL/6J mice were differentiated into osteoblasts. (A) Gene expression of iron transport molecules was assessed. ∆Ct values (Ct_target_ − Ct*_Actb_*) were used to visualize the expression. (B) Osteoblasts were differentiated for 21 d with concomitant treatment of 50 μM of FAC, hemin or holo-Tf. Alizarin Red S staining was used to visualize mineralization. Data represent mean ± SD. Statistical analysis was performed using the Student’s *t*-test. Each dot represents an individual mouse. ^***^p < .001.

Since Tfr1-deficiency did not majorly alter osteoblast iron status and differentiation, we assessed the expression of other transporters associated with iron uptake, including Zip8 and Zip14 for NTBI uptake, Scara5 (ferritin receptor), Hcp1 (heme uptake), and Cd44 (iron bound to hyaluronate) in osteoblasts derived from C57BL/6J mice. We found that Zip8 (encoded by *Slc39a8*) and Zip14 (encoded by *Slc39a8*) as well as Scara5, Hcp1 (encoded by *Slc46a1*) and Cd44 were expressed in osteoblasts at comparable levels as Tfr1 (Figure 4A). Next, we wondered how iron overload induced by transferrin-bound iron and other forms of NTBI affects osteoblast function. Therefore, we treated differentiated osteoblasts from C57BL/6J mice with 50 μm of either FAC, hemin, or holo-transferrin (holo-Tf). FAC as well as hemin significantly reduced the mineralization capacity of osteoblasts, while holo-Tf had no significant impact (Figure 4B).

These results show that osteoblasts express a variety of iron transport molecules apart from Tfr1 and further suggest that excess of NTBI forms may have a more profound impact on osteoblast differentiation.

## Discussion

In the present study, we aimed to investigate the importance of Tfr1 for bone homeostasis and bone cell function. We report increased bone mass and decreased bone turnover in mice deficient of Tfr1 in osteoprogenitors at varying anatomical sites between sexes. Transferrin receptor 1 deficiency can however not mitigate Ovx-induced bone loss. On a cellular level, Tfr1-deficiency does not majorly impact osteoblast function.

Osteoclast differentiation and function are highly dependent on iron.^21^^,^^22^ The partial deficiency of Tfr1 in Tfr1^fl/fl^;LysM:cre^+/−^ did however not suffice to induce bone alterations. Our findings therefore agree with a previous study reporting no bone phenotype in Tfr1^fl/fl^;LysM:cre^+/−^ mice.^24^ Only when the authors used 2 copies of the cre-recombinase (*Tfrc^flox/flox^;Lyz2^Cre/Cre^*), which resulted in a >80% deletion of Tfr1, a higher bone volume was observed in trabecular bone mass in long bones of female mice at 10- and 24-wk of age.^24^ Even though Tfr1 was highlighted as a major iron acquisition pathway in osteoclasts leading to a marked reduction in intracellular iron content in vitro, the in vivo phenotype was not consistent across different bone regions (femur vs spine) or sex (no difference in males).^24^ Das et al. could partially rescue the cytoskeletal phenotype and osteoclastogenesis of Tfr1-deficient osteoclasts by the addition of hemin but not FAC, highlighting heme uptake as an alternative iron acquisition pathway in the absence of Tfr1.^24^

In osteoblast-specific Tfr1-deficient mice, site- and sex-dependent differences in bone mass were observed with a more pronounced bone phenotype in the appendicular skeleton in females and the axial skeleton in male mice. The data highlight sex as a biological variable, which is now obtaining more and more significance.^32^ Not at least, estrogen, for example, is a known regulator of bone as well as iron homeostasis.^33^^,^^34^ Also, in Tfr1^fl/fl^;Osx:cre^+/−^ mice, the question arises whether a more efficient knockdown of Tfr1 in osteoblasts could have resulted in a more pronounced phenotype. Nonetheless, the deficiency of Tfr1 resulted in a mild bone phenotype. Overall, trabecular bone volume was slightly increased, while bone turnover seemed to be slowed down by deficiency of Tfr1 in osteoprogenitors. Since bone resorption and formation are tightly coupled, one explanation for the increased bone mass could be impaired crosstalk of osteoblasts and osteoclasts resulting in decreased osteoclast activity. No difference in the number of osteoclasts was found between genotypes. The expression levels of *Rankl* and *Opg* in femora were both increased in the absence of Tfr1, leading however to no alteration in the Rankl/Opg Ratio. The increase in Rankl and Opg hints toward an imbalance in the signaling between osteoblasts and osteoclasts in response to Tfr1-deficiency. These results suggest an altered interaction of osteoblasts and osteoclasts but cannot be conclusively addressed in the present study. Finally, it should be noted that the knockdown efficiency of Tfr1 in osteogenic cells was also incomplete (~50%). Thus, as in the case of the LysM:cre, it may be possible that a more efficient knockdown of Tfr1 might also lead to more pronounced changes in osteoblast function and bone metabolism. However, absolute deficiencies are difficult to obtain using the Cre-loxP system.

Bone formation by osteoblasts is an energy-demanding process, requiring iron as a cofactor for metabolic processes but also for the process of hydroxylation of amino acid residues during collagen synthesis.^35^ Since Tfr1-deficient osteoblasts showed reduced levels of cytosolic iron but no reduction in protein levels of ferritin, cellular functions are likely maintained, resulting in only minor alterations in mineralization. Since we did not observe a pronounced iron phenotype in osteoblasts deficient for Tfr1, we hypothesize that also other iron transporters compensated for the lack of Tfr1. Iron can enter cells in different forms apart from being bound to transferrin. Nontransferrin bound iron (ferric iron or iron complexes) has been shown to enter cells via Zip8, Zip14, Dmt1, or calcium channels. Iron in the form of ferritin can be taken up by Scara5 or Timd2.^1^ Recently, Cd44 facilitates the endocytosis of iron bound to hyaluronate, a major glycosaminoglycan of the extracellular matrix, which has been described in the context of iron uptake^36^ and presents a possible option in bone since glycosaminoglycans are a major organic compartment of the bone matrix.^37^ We were able to show that besides Tfr1, osteoblasts express transporter molecules for NTBI, heme as well as protein-bound forms of iron (Scara5, Hcp1), which could possibly be involved in iron uptake under Tfr1-deficient conditions. Moreover, our group has previously shown that treatment of osteoblasts with FAC induced a greater reduction in matrix mineralization than holo-Tf.^38^ Furthermore, another study showed that high concentrations of holo-Tf did not influence osteoblast differentiation but improved deferoxamine-reduced mineralization and differentiation.^39^ We also found FAC and hemin to have a greater impact on osteoblast mineralization than holo-Tf, further highlighting possible alternative iron uptake strategies apart from Tfr1 and the detrimental effect of iron taken up as NTBI.

Bone homeostasis is highly dependent on balanced iron levels. The bone phenotype of various mouse models in the absence of key regulators of systemic iron metabolism has been described.^21^^,^^23^^,^^40–42^ Our group has previously highlighted the role of Tfr2 (involved in iron sensing, less in iron uptake due to a lower affinity to Tf than Tfr1) in osteoblasts and could show that cell-specific Tfr2-deficiency in osteoblasts results in a high bone mass with increased bone turnover due to alterations in BMP signaling resulting in Wnt activation.^41^ By depleting Tfr1 from osteoblasts, the focus of this study was laid on iron uptake mechanisms in bone cells. Our and previous studies emphasize diverse iron acquisition strategies employed between osteoblast and osteoclasts. Further efforts in studying iron uptake mechanisms in bone allow to improve our understanding of iron-imbalance-mediated bone disease.

In summary, our results indicate that Tfr1 plays a minor role in osteoblast differentiation in vitro, inducing an increase in bone mass in vivo in a sex- and site-dependent manner, most likely due to altered interactions of bone cells.

## Supplementary Material

REVISION_Supplementary_Material_ziaf069

## Data Availability

The data that support the findings of this study are available on request from the corresponding author (M.R.).
