# Supplementary material for: Bone phenotype in male and female mice after knockdown of transferrin receptor 1 in osterix-expressing cells
Source: JBMR Plus. 2025 May 23;9(7):ziaf069. doi: 10.1093/jbmrpl/ziaf069 (PMC12145871; doi:10.1093/jbmrpl/ziaf069)
Supplement: REVISION_Supplementary_Material_ziaf069 [file revision_supplementary_material_ziaf069.docx]

**SUPPLEMENTARY MATERIAL**

**Table S1.** Primer sequences used for RT-qPCR.

| **gene** | **forward** | **reverse** |
| --- | --- | --- |
| ***Acp5*** | acttgcgaccattgttagcc | agagggatccatgaagttgc |
| ***Actb*** | GATCTGGCACCACACCTTCT | GGGGTGTTGAAGGTCTCAAA |
| ***Alpl*** | CTACTTGTGTGGCGTGAAGG | CTGGTGGCATCTCGTTATCC |
| ***Bglap*** | GCGCTCTGTCTCTCTGACCT | ACCTTATTGCCCTCCTGCTT |
| ***Cd44*** | TCCTTCGATGGACCGGTTACC | GTGGAGCCGCTGCTGACATC |
| ***Col1a1*** | ACTGTCCCAACCCCCAAAG | CGTATTCTTCCGGGCAGAAA |
| ***Ctsk*** | AAGTGGTTCAGAAGATGACGGGAC | TCTTCAGAGTCA ATGCCTCCGTTC |
| ***Opg*** | CCTTGCCCTGACCACTCTTA | ACACTGGGCTGCAATACACA |
| ***Oscar*** | TGGCGGTTTGCACTCTTCA | GATCCGTTACCAGCAGTTCCAGA |
| ***Rankl*** | CCAAGATCTCTAACATGACG | CACCATCAGCTGAAGATAGT |
| ***Scara5*** | CCAAAGGTGACCAAGGAAATGAA | CACCAGGCGAATCATTGTGAAAT |
| ***Slc39a8*** | TCAGCGTTGTATCCCTCCA | GTTTGGGCCCCTTCAGAC |
| ***Slc39a14*** | TGGAACCCTCTACTCCAACG | CTGAGGGTTGAAGCCAAAAG |
| ***Slc46a1*** | TACGGATTGCTCTTCCTGTCATT | CATCAACATGGCCAAGCTATTCA |
| ***Tfrc*** | TGCAGATGAAGAAGAAAATGCCG | CCAGTTTCACACACTCCTCTTTT |

**Table S2.** Blood and iron parameters of 12- and 24-week-old Tfr1^fl/fl^;LysM:cre mice

| **Parameter** | **Male** | | | **Female** | | |
| --- | --- | --- | --- | --- | --- | --- |
|  | **Cre-** | **Cre+** | **P-value** | **Cre-** | **Cre+** | **P-value** |
| **Tfr1^fl/fl^;LysM:cre** |  |  |  |  |  |  |
| *12 weeks* | *n = 17* | *n = 11* |  | *n = 15* | *n = 17* |  |
| Body weight [g] | 29.9 ± 2.36 | 29.9 ± 2.88 | 0.973 | 21.5 ± 2.15 | 21.7 ± 1.69 | 0.772 |
| RBC [x10^6^/µL] | 9.4 ± 0.55 | 9.4 ± 0.62 | 0.749 | 9.5 ± 0.49 | 9.5 ± 0.77 | 0.887 |
| HGB [mmol/L] | 9.0 ± 0.61 | 9.0 ± 0.61 | 0.917 | 9.3 ± 0.45 | 9.3 ± 0.67 | 0.991 |
| HCT [L/L] | 0.48 ± 0.03 | 0.48 ± 0.03 | 0.829 | 0.49 ± 0.03 | 0.49 ± 0.05 | 0.881 |
| MCV [fL] | 50.2 ± 1.27 | 51.1 ± 1.22 | 0.116 | 51.4 ± 1.7 | 50.9 ± 1.68 | 0.403 |
| MCH [fmol] | 0.96 ± 0.03 | 0.96 ± 0.02 | 0.491 | 0.98 ± 0.03 | 0.97 ± 0.02 | 0.748 |
| Plasma Fe [µg/dL] | 229.2 ± 95.8 | 244.3 ± 64.7 | 0.662 | 230.5 ± 80.7 | 235.9 ± 71.3 | 0.863 |
| Tf-Saturation [%] | 42.5 ± 14.9 | 50.8 ± 9.2 | 0.139 | 44.2 ± 16.3 | 60.5 ± 11.1 | **0.010** |
| Liver Iron [µg/g of dry tissue] | 207.4 ± 74.3 | 195.9 ± 73.9 | 0.701 | 280.9 ± 93.2 | 393.3 ± 106.2 | **0.004** |
|  |  |  |  |  |  |  |
| *24 weeks* | *n = 15* | *n = 10* |  | *n = 11* | *n = 11* |  |
| Body weight [g] | 30.8 ± 4.5 | 28.8 ± 5.3 | 0.346 | 27.4 ± 4.2 | 26.1 ± 4.6 | 0.524 |
| RBC [x10^6^/µL] | 9.8 ± 0.86 | 9.4 ± 0.70 | 0.208 | 9.7 ± 0.95 | 9.7 ± 0.56 | 0.920 |
| HGB [mmol/L] | 9.0 ± 0.79 | 8.7 ± 0.69 | 0.284 | 9.3 ± 0.87 | 9.2 ± 0.51 | 0.933 |
| HCT [L/L] | 0.49 ± 0.05 | 0.47 ± 0.03 | 0.140 | 0.49 ± 0.05 | 0.49 ± 0.03 | 1.000 |
| MCV [fL] | 50.3 ± 0.97 | 49.7 ± 1.17 | 0.236 | 50.7 ± 1.27 | 50.9 ± 1.05 | 0.718 |
| MCH [fmol] | 0.92 ± 0.02 | 0.93 ± 0.02 | 0.574 | 0.95 ± 0.02 | 0.95 ± 0.01 | 0.981 |
| Plasma Fe [µg/dL] | 275.9 ± 126.9 | 283.7 ± 158.0 | 0.904 | 262.4 ± 118.9 | 210.3 ± 40.6 | 0.205 |
| Tf-Saturation [%] | 43.1 ± 8.24 | 43.0 ± 10.2 | 0.985 | 46.5 ± 9.93 | 51.4 ± 10.5 | 0.300 |
| Liver Iron [µg/g of dry tissue] | 378.6 ± 143,1 | 289.7 ± 114.0 | 0.133 | 745.5 ± 268.5 | 584.6 ± 181.1 | 0.132 |

RBC = red blood cells; HGB = hemoglobin; HCT = hematocrit; MCV = mean corpuscular volume; MCH = mean corpuscular hemoglobin; Tf-Saturation = transferrin saturation. Data represent the mean ± SD. Statistical analysis was performed by Student’s t-test. Significant values (p<0.05) are highlighted in bold text.

**Table S3.** Iron and bone parameters of 12-week-old Tfr1^fl/fl^;Bglap:cre mice

| **Parameter** | **Male** | | | **Female** | | |
| --- | --- | --- | --- | --- | --- | --- |
|  | **Cre-** | **Cre+** | **P-value** | **Cre-** | **Cre+** | **P-value** |
| **Tfr1^fl/fl^;Bglap:cre** |  |  |  |  |  |  |
| *12 weeks* | *n = 19* | *n = 13* |  | *n = 18* | *n = 17* |  |
| Body weight [g] | 25.7 ± 3.58 | 26.1 ± 2.25 | 0.769 | 21.2 ± 0.57 | 20.6 ± 1.98 | 0.495 |
| Hepatic Iron [µg/g of dry tissue] | 265 ± 99 | 296 ± 143 | 0.494 | 419 ± 143 | 389 ± 196 | 0.673 |
| Splenic Iron [µg/g of dry tissue] | 2261 ± 1906 | 1347 ± 258 | 0.192 | 4026 ± 1487 | 3217 ± 1248 | 0.128 |
| Femur - BV/TV [%] | 11.5 ± 2.9 | 10.5 ± 3.8 | 0.396 | 3.6 ± 0.5 | 4.9 ± 2.0 | 0.085 |
| Femur - Tb.N [1/mm] | 4.5 ± 0.3 | 4.6 ± 0.5 | 0.671 | 3.1 ± 0.3 | 3.2 ± 0.3 | 0.492 |
| Femur - Ct.Th [mm] | 0.19 ± 0.01 | 0.18 ± 0.01 | 0.278 | 0.17 ± 0.01 | 0.18 ± 0.01 | 0.390 |
| L4 - BV/TV [%] | 16.4 ± 3.1 | 14.4 ± 2.7 | 0.074 | 14.5 ± 1.5 | 15.4 ± 2.8 | 0.390 |
| L4 - Tb.N [1/mm] | 4.3 ± 0.3 | 4.1 ± 0.2 | 0.136 | 3.9 ± 0.4 | 3.8 ± 0.4 | 0.528 |
| PINP [ng/mL] | 40.9 ± 10.4 | 41.6 ± 8.1 | 0.871 | 30.9 ± 6.6 | 25.5 ± 5.4 | 0.072 |
| TRAcP5b [U/L] | 9.7 ± 1.1 | 8.4 ± 1.8 | 0.084 | 10.7 ± 3.4 | 9.8 ± 3.4 | 0.576 |

BV/TV = bone volume per total volume, Tb.N = trabecular number, Ct.Th = cortical thickness, PINP = N-terminal propeptide of type I procollagen, TRAcP5b = tartrate-resistant acid phosphatase form 5b

**Figure S1. Partial Tfr1 deficiency in osteoclast precursors has no significant impact on bone mass.** Bone volume per total volume (BV/TV) of (A) distal femur and (B) L4 of 12- and 24-week-old male Tfr1^fl/fl^;LysM:cre mice were analyzed by µCT. (C-D) Serum levels of N-terminal propeptide of type I procollagen (PINP) and tartrate-resistant acid phosphatase form 5b (TRAcP5b) were measured. Histological parameters of the fourth lumbar vertebrae were assessed including (E) bone formation rate per bone surface (BFR/BS) and (F) number of osteoclasts per bone perimeter (N.Oc/B.Pm). Bone volume per total volume (BV/TV) of (G) distal femur and (H) L4 of 12- and 24-week-old female Tfr1^fl/fl^;LysM:cre mice and (I-J) 14-week-old females after ovariectomy were analyzed by µCT. Scale bar, 100 µm. Data represents the mean ± SD (n=10-17). Each dot represents an individual mouse. For comparison of two groups, Student’s *t*-test was performed. For experiments with two factors, two-way ANOVA followed (post-hoc Bonferroni) was performed. *p<0.05, ***p<0.001
